# Oral Manifestations in Menopause—A Scoping Review

**DOI:** 10.3390/medicina61050837

**Published:** 2025-05-01

**Authors:** Anca Labunet, Adriana Objelean, Andreea Kui, Laura Rusu, Alexandra Vigu, Sorina Sava

**Affiliations:** 1 Dental Materials Discipline, Department 4—Prosthodontics and Dental Materials, Faculty of Dental Medicine, “Iuliu Hatieganu” University of Medicine and Pharmacy, 400012 Cluj-Napoca, Romania; labunet@yahoo.com (A.L.); dascalu.monica@umfcluj.ro (L.R.); savasorina@elearn.umfcluj.ro (S.S.); 2 Prosthetic Dentistry Discipline, Department 4—Prosthodontics and Dental Materials, Faculty of Dental Medicine, “Iuliu Hatieganu” University of Medicine and Pharmacy, 400012 Cluj-Napoca, Romania; 3 Dental Materials and Ergonomics Discipline, Faculty of Nursing and Health Sciences, “Iuliu Hatieganu” University of Medicine and Pharmacy, 400012 Cluj-Napoca, Romania; alexandravigu@elearn.umfcluj.ro

**Keywords:** menopause, oral health, saliva

## Abstract

*Background and Objectives*: Menopause is a natural physiological process involving hormone production changes, affecting many functions and systems. This scoping review offers a contemporary outlook on oral issues related to menopause, such as saliva production, periodontal and alveolar bone issues, and changes in the microbiome, and it also investigates the effects of hormonal therapy. *Materials and Methods:* A literature search from 2019 to 2024 was conducted according to PRISMA-ScR guidelines. Articles investigating the oral effects of menopause were included. *Results*: A total of 30 studies were covered; 8 focused on salivary alterations, 5 on periodontal issues, 7 on bone, 3 on the microbiome, and 7 on multiple oral problems, showing that xerostomia and altered taste are the most common oral manifestations, followed by indirect causal effects on periodontitis. Many of these alterations can be contained through regular consultations and adequate hygiene. Some alveolar bone changes may occur after menopause and are associated with osteoporosis. *Conclusions*: Postmenopausal women experience notable reductions in salivary flow, pH levels, and taste sensitivity, which are associated with hormonal fluctuations as well as factors such as age, medication use, and treatments for climacteric symptoms. This population is at increased risk for periodontitis, tooth loss, altered taste, lichen planus, candidiasis, and decreased bone mineral density, which also affect the peri-implant area. Osteoporosis and hormonal changes can play a significant role in causing these increased risks. Maintaining proper oral hygiene and consistently monitoring bone health are essential. While changes in the oral microbiome are more heavily influenced by reductions in salivary flow than by menopause itself, hormone therapy may help improve periodontal health by reducing harmful bacteria and fostering a more balanced microbial environment. The intricate impact of hormones on oral health highlights the necessity for further research.

## 1. Introduction

Menopause is a natural biological process that usually happens in a woman’s 40s or 50s [1]. It signifies the end of menstruation, resulting from decreased ovarian follicular activity. Perimenopause refers to the transitional phase that starts with the first symptoms and continues for one year after the last menstrual period. This stage can last for several years and affect physical, emotional, mental, and social health [1]. 

As postmenopausal changes across various systems can alter inflammatory mediators, vascular permeability, and fibroblast growth and differentiation, women experience a wide range of symptoms: vasomotor and genitourinary, increased depression and anxiety, cognition and sleep decline, high risk of bone resorption [2], and oral issues. The most common oral manifestations include burning mouth, reduced salivary flow, altered taste, oral pain, and dental and periodontal decay [3]. However, hormonal changes may not affect every woman, as factors like genetics and prior dental care can help mitigate oral health problems [4].

Burning mouth syndrome, or stomatodynia, primarily affects women and often emerges during perimenopause. It is more commonly observed in postmenopausal women, with the incidence of related symptoms increasing with age in both males and females [5]. Receptors for female sex hormones have been identified in the oral mucosa, salivary glands, and the trigeminal nerve. Therefore, a decline in sex hormones leads to alterations in neuronal reactivity and expression across different nervous system regions. Estrogen has been shown to potentially increase the number of pain receptors and the electrical impulses perceived as pain by the brain. Still, contrasting evidence suggests that estrogen may also exert an analgesic effect by suppressing nerve growth factors [6]. These findings lead us to believe that hormonal levels decreasing after menopause may enhance burning mouth syndrome prevalence in these circumstances.

Severe oral mucosal disorders may also manifest, including candidiasis, pemphigus vulgaris, benign mucosal pemphigoid, lichen planus, and oral ulcers. Furthermore, postmenopausal women may encounter trigeminal neuralgia and atypical facial pain or neuralgia [3]. Fluctuations in estrogen levels and variations in bone mineral density may accelerate alveolar bone loss, thereby impacting periodontal health and potentially leading to tooth loss [3,7].

During menopause, there is a significantly decreased salivary flow rate, pH, and sweet intensity [7,8], which also places postmenopausal women at a greater risk for dysphagia than premenopausal women [9]. Low estrogen levels after menopause may contribute to temporomandibular joint degeneration and accelerate alveolar bone loss [10]. Fluctuating estrogen levels make women more prone to experiencing prolonged temporomandibular joint (TMJ) pain. Research indicates that estrogen plays a protective role in preventing TMJ degeneration, while low estrogen levels increase the risk of TMJ disorders, particularly in postmenopausal women compared to age-matched men. Additionally, newer studies have found that TMD pain tends to decrease when estrogen levels are elevated, such as during pregnancy. The findings suggest that high estradiol levels enhance protease activity and reduce the production of extracellular matrix components essential for maintaining a healthy TMJ disk [10].

Limited knowledge of oral hygiene and infrequent dental visits among women in perimenopause or menopause stages contribute to poor oral health outcomes. Healthcare providers may sometimes demonstrate inadequate awareness when advising on the importance of regular dental check-ups and informing women about oral health changes during menopause. Additionally, there is a lack of clear guidelines to support tailored care for women and guide providers in their practice [11].

During the menopausal transition, around 50% to 75% of women experience vasomotor symptoms. Estrogen hormone therapy is the primary treatment for these symptoms, though nonhormonal options can also be effective. While there are some contraindications of estrogen therapy related to cardiovascular disease, the overall risks of stroke, venous thromboembolism, and breast cancer remain fairly low [12]. Recent research indicates that hormone therapy is safe for younger women, those within ten years of menopause, and those using low doses for short periods to relieve menopausal symptoms. Transdermal treatments, newer low-dose oral therapies, and selective estrogen receptor modulators may also carry lower risks and could be viable options for women [13].

This scoping review aims to offer an updated outlook on oral issues related to menopause, including studies published in the last 5 years. The main research sections include saliva production, periodontal and alveolar bone issues, and changes in the microbiome. Moreover, we wish to verify two hypotheses: (1) hormonal replacement therapy positively affects oral manifestations and (2) age, rather than hormonal changes, is an underlying factor for oral manifestations.

## 2. Materials and Methods

This literature review was conducted following the PRISMA guidelines (Preferred Reporting Items for Systematic Reviews and Meta-analysis) [10] and the research question was defined through the PICOT format (population, intervention, comparison, outcomes, and time) [11]. **P**: women at menopause; **I**: oral issues analysis; **C**: comparison between oral topics before and after menopause; **O**: influence of menopause on oral manifestations; and **T**: studies published in the past 5 years.

### 2.1. Information Sources and Search Strategy

The search was conducted by two reviewers (AL and AO) from 2 November to 10 November 2024, utilizing three bibliographic databases: Medline (PubMed), Scopus, and Embase. Four main search concepts were established (Table 1), along with a set of keywords and search terms, including MeSH terms, ensuring consistent use across all databases. Detailed search combinations for each database are provided in Table 2. Besides these database searches, a manual search was also performed, reviewing references from multiple studies to pinpoint additional relevant and eligible studies.

### 2.2. Eligibility Criteria

#### 2.2.1. Inclusion Criteria

1. In vivo studies were performed on a female population, including a group undergoing menopause, compared to other population groups.
2. Articles focusing on oral manifestations in menopause, such as saliva alteration, microbiome, bone restructuring, and periodontal involvement.
3. Studies published in English and completed between 2019 and 2024.

#### 2.2.2. Exclusion Criteria

1. Studies that focus only on postmenopausal women, without comparison to premenopausal women or those with normal hormonal levels.
2. Studies that do not report on oral issues associated with menopause.
3. Articles published in languages other than English that are older than 5 years.

### 2.3. Data Extraction and Method of Analysis

Data extraction utilized a standardized form, and all information was documented in an Excel table (v.15.17—Microsoft, Redmond, DC, USA). The extracted data encompassed bibliographic details (authors, title, publication year, and journal), study design and methodology, sample size, outcomes pertaining to oral characteristics, key findings, and conclusions.

To maintain consistency and reduce bias, two reviewers independently extracted data, addressing any discrepancies through discussion or, if necessary, by involving a third reviewer. A standardized template was used to record key details of the studies, such as the purpose, sample, groups, and results pertinent to the review objectives. The primary reviewers conducted the initial data extraction, which was later checked for accuracy and completeness by two other investigators.

## 3. Results

### Data Collection

**Figure 1:**
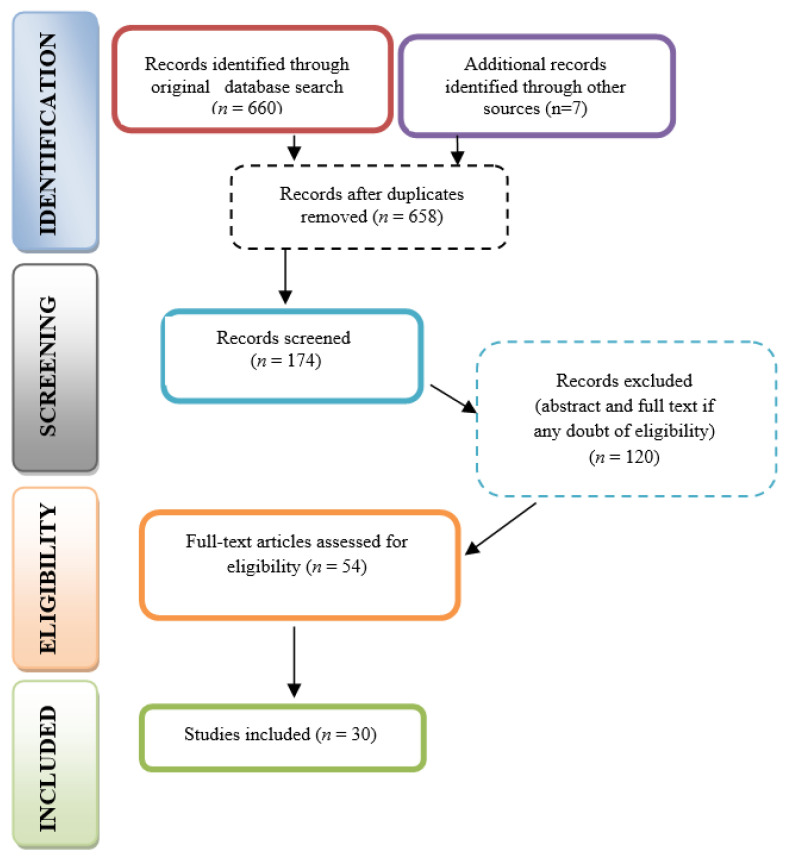
PRISMA flow diagram.

The initial search strategy, outlined in Table 1 and Table 2, yielded 667 articles. After removing duplicates, 658 unique records remained. Following the application of inclusion and exclusion criteria, 174 articles were selected for screening. During the first phase, titles and abstracts were assessed for relevance to the research question, narrowing the pool to 54 articles. Of these, 48 underwent a full eligibility review. Any discrepancies were resolved through discussion and, when necessary, consultation with a third and fourth reviewer. In the end, 30 studies were included in the final review. The PRISMA flow diagram illustrates the selection process and inclusion criteria (Figure 1).

Seven studies explored oral symptoms using a scoping approach, offering a broad overview of the changes associated with menopause. Nine studies focused on quantitative and qualitative alterations in saliva, with Table 3 presenting the most important findings. Five articles in this review specifically addressed periodontal issues; their conclusions are presented in Table 4. Seven studies are included in Table 5, showing osseous alterations, and just two studies researched microbiome changes.

## 4. Discussion

### 4.1. Saliva Quantitative and Qualitative Alterations

Most studies on oral manifestations in menopause focused on salivary flow—eight studies. The main focus was saliva production, as reduced salivary function or flow can lead to the accumulation of dental plaque, which may result in caries and potentially gingivitis [33].

A study evaluated the differences in the physico-chemical properties of saliva between menopausal and premenopausal women, revealing statistically significant decreases in salivary flow rate, lysozyme, and ionized calcium concentrations post-menopause. However, no significant differences were observed in pH levels or concentrations of lactoferrin and immunoglobulin A. The salivary flow rate in menopausal women was significantly lower than in premenopausal women, which may elevate the risk of certain pathological changes in the oral cavity [14,32].

By focusing on taste perception and zinc levels’ influence on taste in premenopausal and postmenopausal women, researchers found that the postmenopausal group had reduced sensitivity to sucrose and quinine hydrochloride, potentially affecting eating habits. However, the taste perception of sodium chloride and citric acid remained unchanged. Contrary to most studies, salivary flow rates in postmenopausal women up to age 60 were found to be within the normal range. Zinc levels were also normal, indicating that it does not significantly influence taste perception, leading researchers to conclude that taste impairment in postmenopausal women is likely a multifactorial phenomenon. However, this study was performed on a total of 60 participants, with no clear criteria being mentioned on the selection of the postmenopausal group [15].

Soundarya et al. found that postmenopausal women had low salivary estradiol levels and high salivary calcium levels. The study revealed that as salivary estradiol levels decreased, xerostomia scores and salivary calcium levels increased. Additionally, higher salivary calcium levels were associated with increased periodontal disease scores [17,19].

Krupa et al. found a significant association between menopausal duration and salivary flow rate. However, menopausal duration did not significantly correlate with tooth loss or oral health-related quality of life [16].

Multiple factors were significantly associated with xerostomia, including reduced salivary flow linked to increased age, number of medications, use of psychotropic drugs, hormone replacement therapy, tongue-burning sensations, and nasal dryness. Additionally, treatments for climacteric symptoms, insomnia, headaches, and nasal dryness were significantly associated with xerostomia. In conclusion, xerostomia is closely linked to climacteric symptoms and their treatment, potentially impacting oral health-related quality of life in perimenopausal women [18].

The number of menopausal symptoms was a variable correlated with xerostomia, taste disturbances, burning mouth, and having more than two oral sensory complaints. The volume of unstimulated saliva was significantly lower in participants experiencing xerostomia, taste disturbances, burning mouth, or multiple oral sensory complaints. Therefore, oral sensory complaints were linked to the number of menopausal symptoms [20].

After examining several inflammation-related cytokines and adjusting for factors like body mass index (BMI), periodontal health, and self-reported physical and emotional well-being, no significant differences were found in salivary cytokine levels between regularly menstruating premenopausal and postmenopausal women. Factors such as BMI, perceived health, serum cytokine levels, and periodontal parameters appear to have minimal impact on these levels in postmenopausal women. Researchers in one study concluded that age may be a more substantial confounding factor [34].

Managing menopausal symptoms may reduce these complaints, thereby improving the quality of life for perimenopausal women [20], specifically with hormone replacement therapy [3].

Our research shows that salivary flow, pH levels, and sweet intensity in postmenopausal women are significantly decreased. These results are consistent with several previous reviews [3,7,8,9,33,35,36]. Reduced salivary flow is a common finding in postmenopausal women and is associated with an increased risk of oral conditions such as xerostomia, caries, and periodontal disease. Taste disturbances and oral sensory complaints in postmenopausal women appear to be multifactorial, linked to both hormonal changes and the number of menopausal symptoms experienced. While hormonal shifts significantly impact saliva composition and flow, other factors such as age, medication use, and climacteric symptom treatments also play a critical role in oral health outcomes during menopause.

### 4.2. Periodontal Issues

Five articles included in this review focused on periodontal issues.

In a study performed in 2021, a premenopausal group showed lower mean scores for plaque index, gingival index, calculus index, pocket probing depth, and clinical attachment loss compared to the postmenopausal group. Consequently, postmenopausal women appear to be more susceptible to periodontitis. Still, regular dental consultations, good oral hygiene practices, and hormone replacement therapies may help mitigate the effects of hormone levels decreasing [21]. Confirming these findings, a study including a large, nationally representative population of South Korea, found that severe periodontitis was associated with factors such as menopausal age, reproductive lifespan, number of pregnancies and abortions, age at first birth, and breastfeeding duration. However, no association was found between severe periodontitis and the use of oral contraceptives or hormone replacement therapy [23]. In contrast, a study on a smaller sample of women found that tooth loss was significantly higher in postmenopausal women compared to regularly menstruating women after adjusting for age. However, there was no significant difference in the prevalence of periodontitis between the two groups. Among postmenopausal women, the prevalence of periodontitis was linked to fewer daily toothbrushing sessions. Additionally, low mood appeared to be associated with both periodontitis and reduced frequency of daily brushing [24].

A study investigating the relationship between endogenous sex hormone levels and tooth loss due to periodontitis in healthy middle-aged to older men and postmenopausal women found that a higher androgenic hormone profile in postmenopausal women was linked to an increased prevalence of tooth loss, even after adjusting for cardiometabolic risk factors [22].

Salivary alkaline phosphatase levels may serve as an early indicator of periodontal disease, as they are associated with changes in bone metabolism. This metabolic balance may be disrupted in postmenopausal women, resulting in clinical attachment loss and tooth loss due to heightened bone resorption [25].

In conclusion, postmenopausal women show greater susceptibility to periodontitis and tooth loss, likely due to hormonal changes and associated bone metabolism alterations. Factors such as menopausal age, reproductive history, and oral hygiene habits significantly influence periodontal health, while the impact of hormone replacement therapy remains inconclusive. Biomarkers like salivary alkaline phosphatase and hormone profiles may serve as useful indicators for assessing periodontal risk in postmenopausal women.

### 4.3. Bone Structure Modifications

A study performed on more than 2000 South Korean women found that osteoporosis was associated with periodontal disease, with the strongest association observed among women in the menopausal transition stage, 0–4 years post-menopause (Lee, 2022, [29]). Additionally, men and postmenopausal women with osteoporosis had higher decayed, missing, and filled teeth (DMFT) indexes compared to those with normal bone mineral density. A correlation was also observed between the DMFT index and bone mineral density in older men and postmenopausal women [30].

Another study suggests that periodontal disease alone does not directly increase the risk of bone loss, but the two conditions may share similar risk factors. Besides age, BMI, and a history of osteoporotic fractures, factors such as radiologically assessed bone density or oral health were not significant predictors of bone loss [26].

Aiming to assess changes in alveolar bone levels around osseointegrated dental implants over two years in premenopausal and postmenopausal women, researchers found a statistically significant increase in marginal bone loss among postmenopausal women. These findings suggest clinicians should stress the importance of oral hygiene maintenance in postmenopausal women to support peri-implant health [27]. Calprotectin level may be a predictor of bone mineral density loss in postmenopausal women with chronic periodontitis [31].

By comparing the groups over and under 50 years of age, researchers showed a lower mean thickness of the crestal cortical bone in the older subjects. However, the crestal cortical bone was only significantly thinner in the posterior maxilla [28].

Another study found that postmenopausal women exhibited significantly higher mean osteocalcin levels and lower bone mineral density than premenopausal women. Bone mineral density significantly and negatively correlated with oral disease status and osteocalcin levels. The two study groups observed a significant difference in the mandibular cortical index [32].

In summary, postmenopausal women, especially those in the early years of menopause, show a strong association between osteoporosis and periodontal disease, with higher rates of tooth decay, loss, and reduced bone mineral density. Bone loss around dental implants and the thinning of the crestal cortical bone are more prominent in postmenopausal women, indicating the importance of maintaining oral hygiene and monitoring bone health in this population. Biomarkers such as calprotectin and osteocalcin may serve as useful indicators of both periodontal disease severity and systemic bone density loss in postmenopausal women.

### 4.4. Microbiome Alteration

Few studies focus on microbiome changes in postmenopausal women. A correlation between a modified oral microbiome and salivary flow rates is present in postmenopausal women. Changes in the oral environment may influence the host–microbial balance, as the salivary microbiome was similar in women with and without Sjögren’s syndrome. However, hyposalivation revealed two distinct bacterial population clusters, indicating that local ecological disturbances could be a driving factor in microbiome changes [37]. Moreover, hormone therapy was linked to a more favorable periodontal profile, with a lower abundance of bacteria such as *T. forsythia* and *T. socranskii*, which are associated with severe periodontal disease. Additionally, hormone therapy was associated with reduced microbial diversity [38].

By studying a microbe population in the oral environment of postmenopausal women, researchers found that the changes were not linked to a dysbiotic shift in the subgingival microbiome, despite findings showing more significant attachment loss in postmenopausal women compared to premenopausal women. Neither decreased estradiol levels nor increased attachment loss during menopause were associated with changes in species abundance or microbiome dysbiosis [39].

Microbiome changes in postmenopausal women appear to be influenced more by salivary flow alterations than by menopause itself. Despite increased attachment loss in postmenopausal women, significant shifts in subgingival microbiome composition or dysbiosis were not observed, indicating complex and multifactorial interactions. Hormone therapy in postmenopausal women may contribute to a healthier periodontal profile.

### 4.5. Several Oral Symptoms

Seven studies investigated oral symptoms in a scoping manner, and their findings provided a general image of menopausal changes.

A study including 90 women in a postmenopausal state, divided into three age-based groups, found the highest prevalence of oral manifestations in patients aged 60 to 70 years. Overall, 76.6% of patients experienced oral mucosal changes, with 20.2% reporting xerostomia, 15.9% periodontitis, 13% altered taste, 8.6% lichen planus, and 7.2% reporting oral candidiasis or space infection [40].

By investigating correlations between oral disorders and serum estrogen levels in postmenopausal women, Ojo et al. found no statistically significant association. However, chronic periodontitis was the most common condition, affecting 54.3% of postmenopausal women, compared to 37.1% of premenopausal women. Hyposalivation was reported in 14.3% of postmenopausal women, compared to 5.7% in premenopausal women. Notably, all postmenopausal women with chronic periodontitis had relatively high estradiol levels, compared to 38.5% of premenopausal women [41].

Other oral manifestations apparently linked to lower hormone levels were found in some studies, including a higher prevalence of females’ self-perceived halitosis [42], satisfaction levels, and occlusal force in complete dentures; there were higher levels in the group of premenopausal women than in postmenopausal women [43], with a higher prevalence of recurrent aphthous stomatitis [44].

Late postmenopausal patients with burning mouth syndrome exhibited increased tongue sensitivity to noxious thermal stimuli. This finding supports the hypothesis that hormonal changes, particularly in the trigeminal somatosensory system, may influence oral pain perception [45].

Only one study was found to contradict some of the previously noted findings. They revealed no significant differences in oral health, taste perception, and nutritional and emotional status between postmenopausal and premenopausal women, but the two study groups only included 30 participants each [46].

A high prevalence of oral mucosal changes was observed among postmenopausal women, particularly indicating a strong trend in symptom occurrence. Common conditions include xerostomia, periodontitis, altered taste, lichen planus, and candidiasis. Chronic periodontitis appears to be the most frequently reported oral condition in postmenopausal women, with a higher prevalence compared to premenopausal counterparts. This suggests a potential link between menopausal status and periodontal health, even though serum estrogen levels did not show a statistically significant correlation in some studies. While one study reported no significant correlation between estrogen levels and oral disorders, others indicated nuanced interactions—for example, all postmenopausal women with chronic periodontitis had relatively high estradiol levels. This highlights the complexity of hormonal influence on oral health and the need for further investigation. One small-scale study found no significant differences between pre- and postmenopausal women in oral health and related factors, emphasizing the variability in findings and the importance of sample size and methodological rigor in future research.

### 4.6. Limitations of the Study and Further Research

This scoping review presents itself with a series of limitations. Firstly, the heterogeneity of studies varies in design, sample size, population, and methodology, rendering comparisons difficult. Secondly, some diagnostic criteria of certain oral issues and age groups in pre-/postmenopausal women were not clearly stated in the methodology, thus affecting the reliability of conclusions. Thirdly, this review is not a meta-analysis and does not provide a statistical synthesis of findings, limiting the strength of conclusions. Findings from specific populations may not be applicable to broader, more diverse populations, as many articles only included women in a certain geographical area or women presenting in the hospital for certain complaints. Most studies primarily focus on differences between premenopausal and postmenopausal women, with limited comparison across sexes.

Further research should include studies on oral issues in both sexes in different age groups and self-perceived versus objective manifestations. There are interesting preliminary findings linking psychological status to oral complaints. Hormonal replacement therapy effects on these problems are not clarified, lacking data on appropriate dosage or type. The initial research reviewed shows a positive influence in some aspects.

## 5. Conclusions

Postmenopausal women experience significant decreases in salivary flow, pH levels, and taste sensitivity, which are linked to both hormonal changes and other factors like age, medication use, and treatment for climacteric symptoms. They are more prone to periodontitis, tooth loss, altered taste, lichen planus, candidiasis, and reduced bone mineral density, with osteoporosis and hormonal changes contributing to these oral health risks. Oral hygiene and regular monitoring of bone health are crucial in this population. Changes in the oral microbiome of postmenopausal women are more strongly influenced by salivary flow alterations than by menopause itself, though hormone therapy may help improve periodontal health by reducing harmful bacteria and promoting a healthier microbial environment.

As for the hypotheses of our study, within the limitations of this scoping review, we conclude that isolating chronological age as a direct contributor to oral manifestations remains challenging, as aging varies in different populations and is influenced by numerous factors. Secondly, hormone replacement therapy has positive effects on salivary changes and the oral microbiome, but no specific data on the effects on periodontal issues and oral bone loss have been found.

The intricate impact of hormones on oral health highlights the necessity for further research. There is considerable variability in the findings, emphasizing the need for larger sample sizes and more rigorous methodologies in future studies.

## Figures and Tables

**Table 1:** Search concepts.

Concept	Keywords and MeSH Terms
Menopause AND dental health	“menopaus*” [Tw] AND “dent*” [Tw] AND “health” [Tw]
Menopause AND oral health	“menopaus*” [Tw] AND “oral” [Tw] AND “health” [Tw]
Menopause AND oral	“menopaus*” [Tw] AND “oral” [Tw]
Menopause AND dental	“menopaus*” [Tw] AND “dent*” [Tw]

**Table 2:** Search combinations per database.

Database	Search Terms and Combinations
PubMed Embase Scopus	“menopaus*”[Tw] AND (“dent*” [Tw] AND **“health**” [Tw]) OR (“**oral**” [Tw] AND **“health**” [Tw]) “menopaus*” [Tw] AND “**oral**” [Tw] OR “dent*” [Tw]

**Table 3:** Articles on salivary alterations (sgn = statistically significant).

No.	Authors, Year Published.	Analyzed Oral Issues	Conclusions
1	Agrawal, Hande, Reche, and Paul [7]	salivary stream rate, pH, gustatory function	postmenopause—sgn (statistically significant) decreased salivary flow rate and pH, decreased sweet intensity
2	Cydejko, Kusiak, Grzybowska, Kochańska, Ochocińska, Maj, and Świetlik [14]	physicochemical properties of saliva	salivary flow rate, lysozyme, ionized calcium concentrations lowered in postmenopause; no differences in pH, lactoferrin, and immunoglobulin A
3	Divya Harika, Garlapati, Badam, Gone, Aiman, Rajani, Kataram, Kulkarni, Manne, and Bontha [15]	taste alterations, salivary flow rate, zinc levels in pre/postmenopause	reduced perceptions of sucrose and quinine hydrochloride, taste impairment is multifactorial; salivary flow rates, zinc levels within normal range postmenopause up to 60 years of age
4	Krupa, Thippeswamy, Chandrashekar, and Thetakala [16]	salivary flow rate, tooth loss, and oral health-related quality of life (OHRQoL)	sgn association menopausal duration—salivary flow rates, tooth loss, and OHRQoL—multifactorial
5	Mishra, Haider, Rizwan, Monga, Pritam, and Singh [17]	saliva and dental health	decrease in the salivary pH and flow rate in postmenopause
6	Shinohara, Ito, Takamatsu, Ogawa, Kajii, Nohno, Sugano, Funayama, Katakura, Nomura, and Inoue [18]	factors associated with xerostomia in perimenopause	xerostomia—association with age, polypharmacy, use of psychotropic medications, hormone replacement therapy, burning sensations of the tongue, nasal dryness, medicine for climacteric symptoms, insomnia, headaches
7	Soundarya, Massillamani, Kailasam, Jayashree, Narmadha, and Sornaa [19]	salivary estradiol, salivary calcium, oral dryness in postmenopause	postmenopause: salivary estradiol levels; low, salivary calcium levels; high, as salivary estradiol levels decreased; increased xerostomia and salivary calcium, thus higher periodontal disease scores
8	Taga, Ito, Takamatsu, Ogawa, Funayama, and Inoue [20]	factors in perimenopausal women’s oral sensory complaints	oral sensory complaints associated with number of menopausal symptoms; management of menopausal symptoms leads to improvements

**Table 4:** Articles focused on periodontal issues (sgn = statistically significant).

No.	Authors, Year Published.	Analyzed Oral Issues	Conclusions
1	Agrawal, Ahmed, Soorgani, Naik, Reddy, and Medabalmi [21]	mean plaque index, gingival index, calculus index, pocket probing depth, clinical attachment loss scores	postmenopausal women are more susceptible to periodontitis
2	Doughan, Chehab, Doughan, Lima, and Michos [22]	endogenous sex hormone levels/periodontitis in middle-aged to older men, post-menopausal women	higher androgenic sex hormone in post-menopausal women—increased prevalence of tooth loss
3	Romandini, Shin, Romandini, Laforí, and Cordaro [23]	hormone-related events and periodontitis in postmenopause	severe periodontitis related to menopausal age, reproductive life length, number of pregnancies/abortions, first birth age and breastfeeding, negative to oral contraceptive and hormone replacement therapy
4	Yakar, Türedi, Emingil, Şahin, Köse, Silbereisen, and Bostanci [24]	periodontal health versus emotional and physical well-being in pre-/postmenopause	association periodontitis—‘’depressed mood’’ indifferent of menopausal status
5	Singh, Verma, Sharma, Gupta, Jha, and Priyank [25]	overall periodontal status and alkaline phosphatase levels in the saliva of females in their pre- and postmenopausal ages.	salivary alkaline phosphatase—sgn differences pre-/postmenopause, decline in periodontal status postmenopause, several factors involved

**Table 5:** Articles on bone issues (sgn = statistically significant).

No.	Authors, Year Published.	Analyzed Oral Issues	Conclusions
1	Ahmad, Tahir, Nauman, Gupta, Gewelber, Batra, and Izuora [26]	association oral health—bone diseases, influencing factors	postmenopause: older age, low BMI, and prior fractures predicted bone loss; periodontal disease did not predict bone loss
2	Dhayanithi and Rajasekar [27]	changes in alveolar bone level around osseointegrated dental implants pre-/postmenopause	increase in marginal bone loss among postmenopausal women—statistically significant
3	Ko, Tsai, Fuh, Tsai, Wang, Huang, and Hsu [28]	thickness of crestal cortical bone at prospective dental implant sites pre-/post menopause	older group—lower mean thickness of the crestal cortical bone; only in the posterior maxilla—bone sgn thinner
4	Lee [29]	association osteoporosis—periodontal disease in Korean menopausal women	osteoporosis—associated with periodontal disease, strongest in women in the transition stage, 0–4 years postmenopause
5	Lee and Myong [30]	relationship bone mineral density—dental caries pre-/postmenopause	men and post-menopausal women with osteoporosis had higher DMFT (decay, missing, filled teeth) indexes—correlation
6	Pavithra, Ramesh, Thomas, Kumari, and Sharmila [31]	salivary calprotectin levels, bone mineral density in post-menopause +/− chronic periodontitis	sgn difference in salivary calprotectin, no sgn difference mean bone mineral density between periodontitis/non-perio patients; positive correlation salivary calprotectin levels—bone mineral density in perio patients
7	Venkatesh, Rajora, Sagare, Bagga, Kaur, and Gandhi [32]	plasma osteocalcin levels status of oral disease, alteration in mandibular bone density in pre-/postmenopause	osteocalcin, oral dryness sgn higher postmenopause; bone mineral density negative sgn correlation with oral disease osteocalcin levels.

## Data Availability

The original contributions presented in this study are included in the article. Further inquiries can be directed to the corresponding authors.
